# Therapeutic effects and outcomes of rescue high-frequency oscillatory ventilation for premature infants with severe refractory respiratory failure

**DOI:** 10.1038/s41598-021-88231-6

**Published:** 2021-04-19

**Authors:** Jen-Fu Hsu, Mei-Chin Yang, Shih-Ming Chu, Lan-Yan Yang, Ming-Chou Chiang, Mei-Yin Lai, Hsuan-Rong Huang, Yu-Bin Pan, Ren-Huei Fu, Ming-Horng Tsai

**Affiliations:** 1 Division of Neonatology, Department of Pediatrics, Linkou Chang Gung Memorial Hospital, Taoyüan, Taiwan; 2 Department of Respiratory Therapy, Chang Gung Memorial Hospital, Taipei, Taiwan; 3 School of Business, Executive MBA Program in Health Care Management, Chang Gung University, Taoyüan, Taiwan; 4 Division of Neonatology and Pediatric Hematology/Oncology, Department of Pediatrics, Chang Gung Memorial Hospital, No.707, Gongye Rd., Sansheng, Mailiao Township, Yunlin, Taiwan, ROC; 5 College of Medicine, Chang Gung University, Taoyüan, Taiwan; 6 Biostatistics Unit of Clinical Trial Center, Chang Gung Memorial Hospital, Linkou, Taiwan

**Keywords:** Medical research, Paediatric research

## Abstract

Despite wide application of high frequency oscillatory ventilation (HFOV) in neonates with respiratory distress, little has been reported about its rescue use in preterm infants. We aimed to evaluate the therapeutic effects of HFOV in preterm neonates with refractory respiratory failure and investigate the independent risk factors of in-hospital mortality. We retrospectively analyzed data collected prospectively (January 2011–December 2018) in four neonatal intensive care units of two tertiary-level medical centers in Taiwan. All premature infants (gestational age 24–34 weeks) receiving HFOV as rescue therapy for refractory respiratory failure were included. A total of 668 preterm neonates with refractory respiratory failure were enrolled. The median (IQR) gestational age and birth weight were 27.3 (25.3–31.0) weeks and 915.0 (710.0–1380.0) g, respectively. Pre-HFOV use of cardiac inotropic agents and inhaled nitric oxide were 70.5% and 23.4%, respectively. The oxygenation index (OI), FiO_2_, and AaDO_2_ were markedly increased after HFOV initiation (all *p* < 0.001), and can be decreased within 24–48 h (all *p* < 0.001) after use of HFOV. 375 (56.1%) patients had a good response to HFOV within 3 days. The final in-hospital mortality rate was 34.7%. No association was found between specific primary pulmonary disease and survival in multivariate analysis. We found preterm neonates with gestational age < 28 weeks, occurrences of sepsis, severe hypotension, multiple organ dysfunctions, initial higher severity of respiratory failure and response to HFOV within the first 72 h were independently associated with final in-hospital mortality. The mortality rate of preterm neonates with severe respiratory failure remains high after rescue HFOV treatment. Aggressive therapeutic interventions to treat sepsis and prevent organ dysfunctions are the suggested strategies to optimize outcomes.

## Introduction

In the past two decades, high-frequency oscillatory ventilation (HFOV) has been used as a rescue therapy for neonates with refractory respiratory failure^1,2^. HFOV is used to recruit compromised lung, improve oxygenation through combined high mean airway pressures (MAPs) and less tidal volumes, and eliminate carbon dioxide with few adverse cerebral side effects^3–6^. Although HFOV is theoretically beneficial for lung protection in preterm infants with severe hypoxemia and hypercapnia^7,8^, there are few information regarding how HFOV-treated premature neonates are managed, especially how ventilation settings and adjunctive therapies used before and after use of HFOV would affect the outcomes^9–11^.

Recent large multicenter studies of randomized clinical trial in neonates reported conflicting results when tested the efficacy of HFOV in congenital diaphragmatic hernia (CDH) and respiratory distress syndrome (RDS)^12–14^. Current research interests focused on the comparisons between elective HFOV use and conventional mechanical ventilation (CV) in premature infants^15–19^. However, the indication to initiate HFOV is not standardized and rare studies have investigated the use of HFOV as final rescue for premature infants with cardiopulmonary failure^20,21^. In this study, we aimed to describe the ventilatory management of HFOV-treated premature neonates with severe respiratory failure, to investigate the impacts of dynamic HFOV changes and other organ failures on final mortality, and to assess the use of adjunctive therapies before and during HFOV and the independent risk factors of final in-hospital mortality.

## Methods

### Study design, settings and patients

We conducted a longitudinal, single-center, retrospective cohort study of patients from the respiratory database of neonatal intensive care unit (NICU) in Linkou and Taipei Chang Gung Memorial Hospitals (CGMH), both are tertiary level, university-affiliated academic medical centers in Taiwan. This respiratory database includes 5581 patients admitted in a total of four NICUs in Taipei CGMH (1 NICU) and Linkou CGMH (3 NICUs) between January 2011 and December 2018, and includes physiologic information from bedside monitors and hospital information systems. During the 8-year study period, all consecutive patients with gestational age < 34 weeks and ≥ 24 weeks who received HFOV for severe respiratory failure were screened. The data in the NICU database has been previously deidentified, and the institutional review board of CGMH has approved the study and use of the database for research. The need for informed consent was waived by institutional review board of Chang Gung Memorial Hospital because all patient records/information were anonymized and deidentified prior to analysis. All methods in this study were performed in accordance with the relevant guidelines and regulations.

We identified and enrolled premature infants who experienced severe refractory respiratory failure during hospitalization and those who failed to respond to conventional ventilation. Severe refractory respiratory failure was defined when patients with a PaO_2_/FiO_2_ ratio under 150 on two consecutive arterial blood gas (ABG) samples and/or had an initial oxygenation index (OI) ≥ 20^22^. Poor response to conventional ventilation was defined as failure to decrease PCO_2_ > 10% and/or FiO_2_ > 20% after 1 h treatment by conventional ventilation (CV) at maximal settings, which usually include the rate of 60 per minute, fraction of inspired oxygenation (FiO_2_) of 100%, a peak end expiratory pressure (PEEP) of 5–6 cm H_2_O, and the appropriate peak inspiratory pressure depending on gestational age^23,24^. Exclusion criteria were patients < 24 weeks of gestation, incomplete medical records, and patients with severe congenital anomalies (ex. hydrops fetalis, congenital leukemia, or trisomy 18. etc.)

In our NICU, the initiation of HFOV, management of respiratory failure, the therapeutic strategies and use of adjuvant therapies were all based on the decisions of the attending physicians. HFOV (Dräger Babylog 8000plus ventilator, Lübeck, Germany) was often used when CV failed to improve oxygenation and/or in the presence of severe hypercapnia or barotraumas. HFOV is initially configured according to the guideline, which includes a frequency of 10–15 Hz and delta P 70–100% of the desired pressure to make appropriate chest wall movement. For mean airway pressure (MAP), a 2–3 cmH_2_O higher than previous conventional ventilator setting is usually applied. The initial management goals are to maintain pH 7.35–7.45, a SpO_2_ value of 85–95% and CO_2_ 45–55 cmH_2_O. During HFOV treatment, neonates were usually sedated with fentanyl and/or midazolam but not completely paralyzed by muscle relaxant. In cases with systemic hypotension, cardiovascular inotropic agents, including dopamine and dobutamine, milrinone and/or epinephrine would be used. Inhaled nitric oxide (iNO) (Solmix 1000; Dutch Technical Gas Company, Tilburg, The Netherlands) was used from 20 ppm to a maximum of 80 ppm, based on the clinical practices^25^. Surfactant (beractant, Sruventa; Ross Laboratories, Columbus, PH; one dose = 100 mg/kg) was administered to newborns with meconium aspiration syndrome (MAS) and respiratory distress syndrome (RDS).

### Data collection and definition

We used case report form and collected a daily expanded data set including MV settings, ABG, adjuvant therapies during pre- and post-HFOV use until HFOV-day 120, death, or successfully weaning from HFOV, whichever occurred first. The patient demographics, underlying comorbidities, presence of central venous catheter (CVC) and other artificial devices, use of inhaled nitric oxide (iNO), antibiotics, and cardiac agents, and other ventilatory adjuvant therapies were also recorded. The validity of all patient data was verified by all attending physicians in charge. Bronchopulmonary dysplasia (BPD) was defined based on the diagnostic criteria of the American Thoracic Society^26^. Severity of hypotension was defined and described in the appendix (supplementary Table 1). We also used standard definitions to evaluate multiple organ dysfunctions, including hepatic, neurological, renal and hematological organ dysfunction (see supplementary Table 1) during the first 3 days of HFOV treatment.

We evaluated the therapeutic response to HFOV within the first 72 h, which was defined as follows: (1) good response: the situation of hypoxemia or hypercapnia was successfully treated by HFOV, and the modality is weaned or ceased at 72 h; (2) Non-significant improvement: The condition of hypoxemia was corrected, and the OI has been improved to a certain extent, but the settings of HFOV cannot be weaned within 72 h and is still needed beyond 72 h; and (3) failure: failure to improve on HFOV treatment, and the average OI on the third day was significantly higher than the first day. All the above study designs, settings, patients and data collection were based on a series of study projects supported by the CGMH research foundation^27^.

### Statistical analyses

Variables with parametric distributions were expressed as mean (standard deviation, SD) and continuous variables with non-parametric distributions were expressed as median (interquartile range, IQR). Comparison between pre- and post-HFOV continuous variables and between survivors versus non-survivors were analyzed using paired Student t test and the paired Wilcoxon rank sum tests. Categorical variables were compared with chi-square tests or Fisher exact tests. All *p*-values were two tailed, and *p*-values < 0.05 were considered to be statistically significant.

The primary outcome measure was the final in-hospital mortality after HFOV use. Associations between patients’ demographic, clinical and pre-HFOV ventilation characteristics, HFOV ventilation characteristics within the first 3 days, and laboratory results were tested in univariate analyses, with odds ratio (OR) quantifying the strength of association. Covariates presumed to be associated with final in-hospital mortality based on previous studies and those associated with mortality at P < 0.1 were subsequently entered in multivariable logistic regression models. Multivariate logistic regression analysis by backward stepwise method was used to develop a predictive model and a nomogram of final in-hospital mortality. The discriminatory capacity of the model was determined by calculating the area under the curve (AUC). The calibration of the model was evaluated by using the Hosmer–Lemeshow test. Youden Index was used to find the best cutoff point for sensitivity and specificity of the nomogram^28^. Statistical analysis was performed using SPSS (version 21.0; IBM, Armonk, NY).

### Ethics approval and consent to participate

This study was approved by the institutional review board of Chang Gung Memorial Hospital, and the need for informed consent was waived by institutional review board of Chang Gung Memorial Hospital because all patient records/information were anonymized and deidentified prior to analysis.

## Results

### Patient demographics

**Figure 1:**
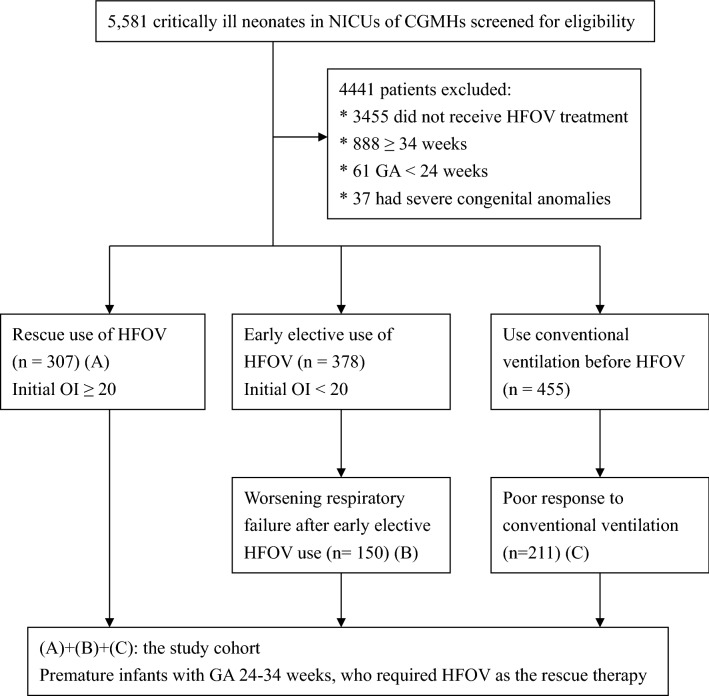
Flow chart and enrollment of the study cohort. HFOV: high frequency oscillatory ventilation; OI: oxygenation index; GA: gestational age; NICU: neonatal intensive care unit; CGMH: Chang Gung Memorial Hospital.

During the study period, a total of 668 premature infants were enrolled (Fig. 1). Among the entire cohort, HFOV was used as the rescue use in 307 (45.9%) patients, who had an initial OI ≥ 20. 211 (31.6%) patients were shifted from conventional ventilation because of poor response to conventional ventilation and/or clinical deterioration. The last 150 (22.5%) patients were enrolled because they experienced worsening respiratory failure during their early elective HFOV use with an initial OI < 20. The median (interquartile range [IQR]) gestational age and birth weight were 27.3 (25.3–31.0) weeks and 915.0 (710.0–1380.0) g, respectively. 61.1% of these patients were male, and most of them (70.4%) were delivered by cesarean section. The most common cause of refractory failure was RDS (62.4%, n = 417), followed by PPHN (22.3%, n = 149), severe sepsis (20.2%, n = 135), pulmonary hemorrhage (9.7%, n = 65), and pneumothorax (9.1%, n = 61). The demographic data of the study cohort are shown in Table 1.

**Table 1:** Baseline characteristics, clinical and biological features at the time of HFOV initiation according to final survival status.

Characteristics	All patients (n = 668)	Final status at discharge post-HFOV use		P value
		Non survivors (n = 232)	Survivors (n = 436)	
Gestational age (weeks), median (IQR)	27.3 (25.3–31.0)	26.5 (25.0–30.0)	27.6 (25.6–31.2)	< 0.001
Birth body weight (g), median (IQR)	915.0 (710.0–1380.0)	793.0 (640–1138.5)	989.5 (760–1520)	< 0.001
Gender, (male/female), n (%)	408 (61.1)/ 260(38.9)	132 (56.9)/100 (43.1)	276 (63.3)/160 (36.7)	0.114
Apgar score at 5 min < 8, n (%)	384 (57.5)	158 (68.1)	226 (51.8)	0.001
Outborn, n (%)	132 (19.8)	42 (18.1)	90 (20.6)	0.262
Caesarean section, n (%)	470 (70.4)	167 (72.0)	303 (69.5)	0.534
Perinatal asphyxia, n (%)	68 (10.2)	33 (14.2)	35 (8.0)	0.015
**Major pulmonary diseases, n (%)**				
Respiratory distress syndrome	417 (62.4)	133 (57.3)	284 (65.1)	0.054
Persistent hypertension of newborn	149 (22.3)	62 (26.7)	87 (19.9)	0.051
Meconium aspiration syndrome	15 (2.2)	3 (1.3)	12 (2.8)	0.226
Congenital diaphragmatic hernia	11 (1.6)	5 (2.2)	6 (1.4)	0.451
Pneumothorax	61 (9.1)	24 (10.3)	37 (8.5)	0.481
Pulmonary interstitial emphysema	14 (2.1)	6 (2.6)	8 (1.8)	0.574
Sepsis	135 (20.2)	68 (29.3)	67 (15.4)	< 0.001
Pneumonia	17 (10.5)	10 (4.3)	7 (1.6)	0.610
Bronchopulmonary dysplasia	46 (6.9)	18 (7.8)	28 (6.4)	0.524
Pulmonary hemorrhage	65 (9.7)	32 (13.8)	33 (7.6)	0.013
**Other cormorbidities, n (%)**				
Patent ductus arteriosus (PDA)	289 (43.3)	83 (35.8)	206 (47.2)	0.005
PDA status post ligation	34 (5.1)	8 (3.4)	26 (5.9)	0.196
Intraventricular hemorrhage^a^	63 (9.4)	26 (11.2)	37 (8.5)	0.268
Congenital heart disease	21 (3.1)	14 (6.0)	7 (1.6)	0.004
Gastrointestinal obstruction	23 (3.4)	10 (4.3)	13 (3.0)	0.379
Others	19 (3.4)	9 (3.9)	10 (2.3)	0.328

Results are presented as n (%) or median (25th, 75th percentiles).

^a^Intraventricular hemorrhage ≥ grade III.

Among the 668 patients, 75.6% of all HFOV treatments were initiated within the first week of life, and 60.0% of the infants received HFOV starting on their first day of life. All these neonates had umbilical arterial catheters or arterial lines to frequently check the blood gas analysis and OI calculation during the most critically ill period. The median duration of HFOV use was 6.0 (3.0–20.0) days, and the median (IQR) duration of intubation with mechanical ventilation was 20.0 (13.0–49.0) days.

### Initial HFOV response and use of adjunctive therapies

Inhaled nitric oxide (iNO) and surfactant were used in 23.4% and 61.7% of cases, respectively. Cardiac inotropic agents were used in 70.5% of all events, and more than half (52.2%) of them required more than one cardiac inotropic agent. Table 2 presents the initial ventilatory settings within the first 2 h after initiation of HFOV. The MAP was increased from 7.8 ± 3.9 cm H_2_O on conventional ventilation (data immediately preceding HFOV) to 13.4 ± 8.5 cm H_2_O at initiation of HFOV (*p* < 0.001). The OI, FiO_2_, and AaDO_2_ at initiation of HFOV were also significantly higher than those on conventional ventilation (all *p* < 0.001, data not shown). Among the entire cohort, more than 80% of patients had an OI greater than 15 preceding initiation of HFOV. After initiation of HFOV, the therapeutic responses were examined at 2 h, 6 h and 12 h after use of HFOV and the average daily OI, AaDO2, and blood gas analysis on the 2nd and 3rd day were also evaluated. Table 3 shows the therapeutic responses after the rescue use of HFOV. We found a significant improvement of oxygenation within the first 6 h after initiation of HFOV, especially in patients on HFOV as rescue use. However, patients who experienced clinical deterioration during HFOV treatment (n = 150) had a significantly higher risk of mortality (*p* < 0.001) and progression to BPD (*p* = 0.032) due to ventilation-related complications or nosocomial infections (Table 3).

**Table 2:** HFOV management and HFOV-related complications during the first 2 days according to final survival status.

Parameter	All patients (n = 668)	Final status at discharge after use of HFOV		P value
		Non survivors (n = 232)	Survivors (n = 436)	
**Ventilation settings, median (IQR)**				
FiO_2_, %	90.0 (60.0–100.0)	100.0 (62.8–100.0)	80.0 (60.0–100.0)	0.001
Mean airway pressure, cm H_2_O	12.0 (10.0–15.0)	12.0 (10.0–15.0)	12.0 (10.0–14.0)	0.105
Delta P (%)^a^	100.0 (73.0–100.0)	90.0 (70.0–100.0)	100.0 (75.0–100.0)	0.001
Frequency, Hz	12.0 (11.0–13.0)	12.0 (11.0–13.0)	12.0 (11.0–13.0)	0.539
PH	7.26 (7.14–7.36)	7.23 (7.07–7.36)	7.27 (7.17–7.36)	0.011
PaCO_2_, mmHg	51.6 (42.0–64.8)	53.9 (42.6–72.0)	50.5 (41.5–62.3)	0.013
PaO_2_, mmHg	48.0 (35.3–66.5)	44.8 (32.2–61.8)	50.0 (37.5–69.0)	0.001
Oxygenation index	12.0 (7.0–20.0)	15.0 (8.0–31.8)	11.0 (7.0–17.0)	< 0.001
AaDO_2_	434 (267–579)	516.5 (280–590.8)	396.5 (259–570)	0.002
**Adjuvant therapies, n (%)**				
iNO use	156 (23.4)	66 (28.4)	90 (20.6)	0.027
Surfactant use	412 (61.7)	132 (56.9)	280 (64.2)	0.079
Dopamine	471 (70.5)	181 (78.0)	290 (66.5)	0.002
Dobutamine	255 (38.2)	122 (52.6)	133 (30.5)	< 0.001
Epinephrine	70 (10.5)	48 (20.7)	22 (5.0)	< 0.001
Milrinone	61 (9.1)	33 (14.2)	28 (6.4)	0.002
Corticosteroid	5 (0.7)	5 (2.2)	0 (0)	0.002

Results are presented as n (%) or median (25th, 75th percentiles).

^a^Delta P* are the expressed as the amplitude on the relative scale from 0 to 100% until the desired pressure or the desired tidal volume is achieved.

**Table 3:** Therapeutic responses and treatment outcomes of premature neonates (total n = 668) treated by high-frequency oscillatory ventilation (HFOV).

Therapeutic responses and treatment outcomes	All patients (n = 668)	Rescue use of HFOV (total n = 518)	Early elective use of HFOV with clinical deterioration (n = 150)	*P* value
OI at 2 h after use of HFOV	19.0 (15.0–34.0)	24.0 (18.0–39.0)	8.0 (6.0–12.0)	< 0.001
OI at 6 h after use of HFOV	12.0 (7.0–20.0)	14.0 (8.0–23.0)	8.0 (5.0–12.5)	< 0.001
OI at 12 h after use of HFOV	14.0 (8.0–23.0)	17.0 (10.0–26.3)	8.0 (5.0–13.0)	< 0.001
Average OI at 2nd day after use of HFOV	15.0 (7.0–30.0)	14.0 (6.0–30.0)	21.0 (15.0–30.0)	< 0.001
Average OI at 3rd day after use of HFOV	12.0 (5.0–27.0)	10.0 (4.0–25.0)	18.0 (8.0–29.0)	< 0.001
Highest OI during the HFOV course	24.0 (18.0–42.0)	22.0 (18.0–48.0)	27.0 (19.0–56.0)	< 0.001
**Therapeutic responses to HFOV**				
Good response	375 (56.1)	303 (58.5)	72 (48.0)	
Non-significant improvement	172 (25.7)	110 (21.2)	62 (41.3)	< 0.001
Failure	121 (18.1)	105 (20.3)	16 (10.7)	< 0.001
Progression to BPD	375 (56.1)	279 (53.9)	96 (64.0)	0.032
Duration of HFOV (days)	6.0 (3.0–20.0)	6.0 (3.0–18.0)	11.0 (4.0–34.5)	< 0.001
Duration of intubation (days)	20.0 (3.0–49.0)	20.0 (4.0–48.8)	19.0 (3.0–49.0)	0.349
Duration of mechanical ventilation (days)	39.0 (10.0–74.0)	35.0 (9.0–72.0)	49.0 (10.5–85.0)	0.078
Duration of hospital stay (days)	75.0 (22.0–113.0)	75.0 (24.0–113.0)	77.0 (14.5–111.5)	0.557
120-day mortality	232 (34.7)	149 (28.8)	83 (55.3)	< 0.001

Results are presented as n (%) or median (25th, 75th percentiles).

OI: oxygenation index; HFOV: high frequency oscillatory ventilation; BPD: bronchopulmonary dysplasia.

### Patient outcomes and predictors of in-hospital mortality

Except for four patients whose family requested transfer to other hospital, all patients’ outcomes were obtained. 455 (68.1%) patients were successfully weaned off HFOV, 414 (62.0%) survived to NICU discharge and 436 (65.3%) were alive at 120 days. Among non-survivors, 100 (39.4%) died within the first week after use of HFOV, and 54 (21.3) died within the first 72 h. 31.9% had use of HFOV for more than 14 days, and 29 (4.3%) had use of HFOV for more than 60 days. The median [IQR] duration of HFOV support, mechanical ventilation and hospital stay for 120-day survivors were 7 [4; 21], 49.5 [14; 74.8], and 95 [52.3; 123.3] days, respectively.

Final survivors had a significant lower rate of perinatal asphyxia, lower percentage to have low Apgar score at 5 min, and were not so extremely preterm and/or low birth body weight when compared with the non-survivors (Table 1). The underlying pulmonary diseases that caused severe respiratory failure were not significantly different between survivors and non-survivors, except sepsis was significantly more common in non-survivors. The mean airway pressure and frequency of HFOV settings at the first 48 h were similar between 120-day survivors and non-survivors. However, non-survivors were administered significantly higher FiO_2_ and delta P, and exhibited higher severity of respiratory acidosis and hypoxemia (Table 2). Of note, most adjuvant therapies, including cardioinotropic agents, iNO, and blood transfusion, were more frequently used during the first 2-days on HFOV in non-survivors than survivors.

**Figure 2:**
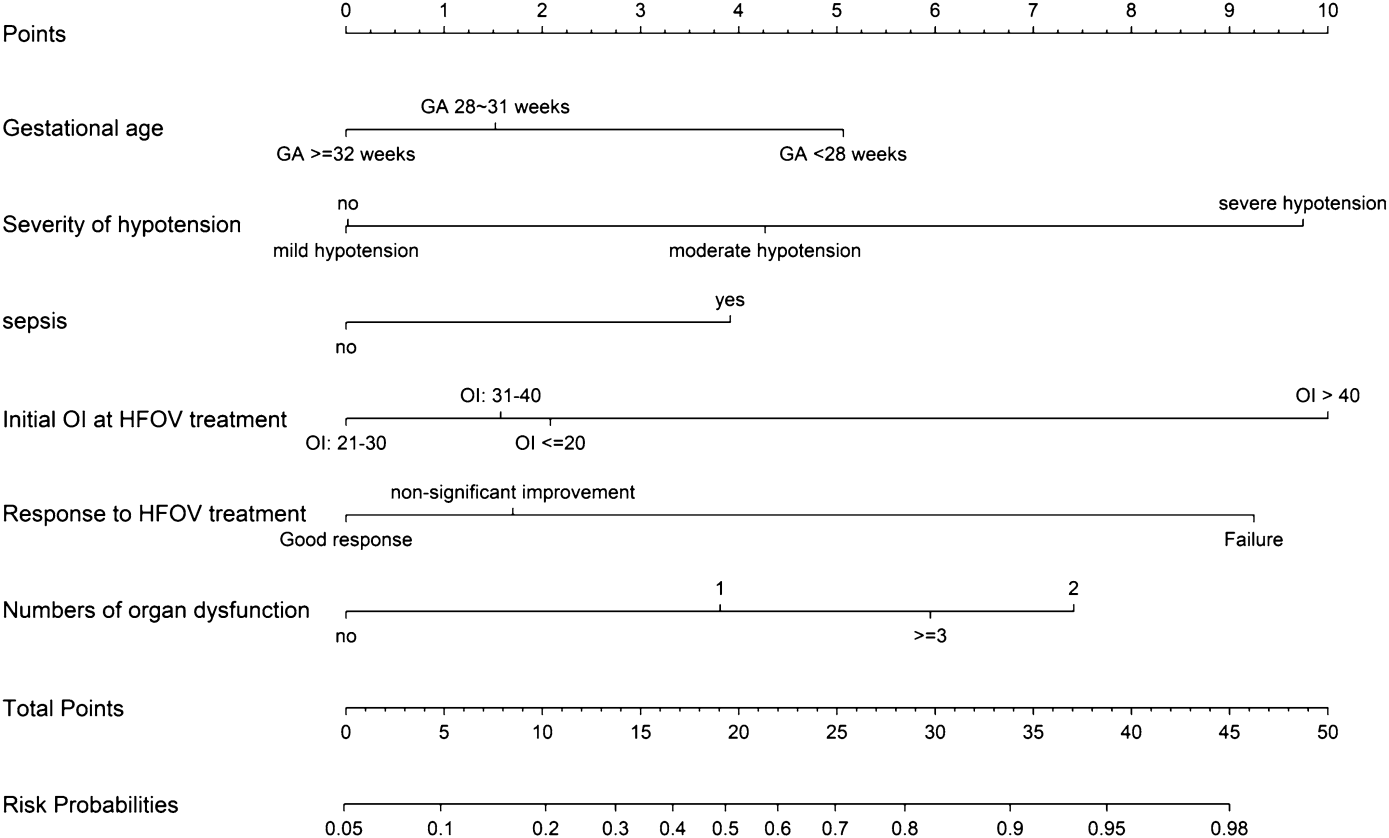
Multivariate stepwise logistic regression-based nomogram to predict the probability of in-hospital mortality for neonates with severe refractory respiratory failure using the following six predictors: gestation age, severity of hypotension, sepsis, initial OI at HFOV treatment, response to HFOV treatment and numbers of organ dysfunction.

Predictive factors of final in-hospital mortality were assessed using univariate analysis and a multivariate logistic model (Supplementary Table 2 and Table 4). Lower birth weight, occurrence of sepsis, severe hypotension, numbers of organ dysfunction, low apgar score (≤ 7) at 5 min, perinatal asphyxia, initial severity of respiratory failure, and initial response to HFOV treatment were jointly selected as the variables to enrolled into the multivariate logistic regression model. No association was found between various pulmonary disease entities and survival in the multivariate analysis. After backward stepwise method, we found six variables, including lower birth weight, occurrence of sepsis, severity of hypotension, numbers of organ dysfunction, initial severity of respiratory failure and response to HFOV treatment were independently associated with final in-hospital mortality (Table 4). The nomogram based on these six independent variables was developed to predict the risk of mortality (Fig. 2), and the calibration curves for the probability were drawn (Supplementary Figure 1). A cutoff point of 0.3 was used based on the Youden index to calculate the sensitivity and specificity of this model, which were 70.6% and 72.8%, respectively. The area under the curve (AUC) for this model is 0.768 (Supplementary Figure 2).

**Table 4:** Risk factors for final in-hospital mortality by multivariate analysis and multivariate stepwise analysis.

Risk factors	Multivariate analysis		Multivariate stepwise regression	
	Adjusted OR (95% CI)	*P* value	Adjusted OR (95% CI)	*P* value
**Gestational age (weeks)**				
< 28 weeks	2.22 (1.27–3.87)	0.005	2.16 (1.24–3.75)	0.007
28–31 weeks	1.29 (0.69–2.40)	0.421	1.27 (0.69–2.34)	0.450
≥ 32 weeks	1 (reference)		1 (reference)	
Low apgar score at 5 min (≤ 7)	1.02 (0.58–1.75)	0.993		
Perinatal asphyxia	1.04 (0.48–2.24)	0.919		
Occurrence of sepsis	1.74 (1.10–2.75)	0.018	1.82 (1.17–2.85)	0.008
**Highest OI during HFOV treatment**				
≤ 30	1 (reference)			
31–40	0.97 (0.52–1.64)	0.746		
41–50	1.25 (0.62–2.55)	0.533		
> 50	1.44 (0.83–2.49)	0.190		
**Initial OI at HFOV treatment**^**a**^				
≤ 20	1 (reference)		1 (reference)	
21–30	0.72 (0.37–1.37)	0.314	0.73 (0.38–1.39)	0.336
31–40	0.92 (0.33–2.53)	0.862	0.93 (0.34–2.55)	0.880
> 40	3.05 (1.15–8.13)	0.026	3.31 (1.27–8.66)	0.015
**Response to HFOV within the first 3 days**				
Good response	1 (reference)		1 (reference)	
Non-significant improvement	1.25 (0.74–2.11)	0.412	1.29 (0.77–2.17)	0.334
Failure	3.64 (1.82–7.27)	< 0.001	4.05 (2.08–7.89)	< 0.001
**Number of organ dysfunction**^**b**^				
No	1 (reference)		1 (reference)	
1	1.73 (0.92–3.23)	0.087	1.77 (1.01–3.08)	0.045
2	2.26 (0.86–5.94)	0.098	2.45 (1.34–4.47)	0.004
≥ 3	2.91 (1.21–6.97)	0.017	3.05 (1.56–5.98)	0.001
**Severity of hypotension**^**c**^				
No	1 (reference)		1 (reference)	
Mild hypotension	0.98 (0.60–1.64)	0.948	1.02 (0.62–1.63)	0.992
Moderate hypotension	1.84 (1.13–3.00)	0.015	1.91 (1.17–3.09)	0.009
Severe hypotension	3.97 (1.94–8.13)	< 0.001	4.37 (2.17–8.80)	< 0.001
**Specific disease entities**				
PPHN	1.44 (0.88–2.37)	0.151		
Pulmonary hemorrhage	1.58 (0.88–2.76)	0.129		
Secondary pulmonary hypertension	3.75 (0.73–19.32)	0.115		
Patent ductus arteriosus	0.83 (0.56–1.22)	0.344		

OI: oxygenation index; RDS: respiratory distress syndrome; PPHN: persistent pulmonary hypertension of newborn; OR: odds ratio, 95% CI: 95% confidence interval.

^a^The average oxygenation index at 2 and 6 h after initiation of HFOV was used.

^b^Number of organ dysfunction: including neurological, renal, hematological, and hepatic dysfunction.

^c^Mild hypotension was defined as patients who required only one cardioinotropic agents (usually dopamine < 10 µg/kg/min); Moderate hypotension was defined as patients who required both dopamine and dobutamine, with only one of them ≥ 10 µg/kg/min to maintain adequate blood pressure; Severe hypotension was defined as patients who required epinephrine and/or more than two cardioinotropic agents (usually dopamine and dobutamine, both ≥ 10 µg/kg/min) to maintain adequate blood pressure.

## Discussion

In this study, we investigated the impact of HFOV rescue use on neonates with severe refractory respiratory failure who can not meet the general neonatal ECMO criteria^29^; i.e. gestational age ≥ 34 weeks or birth weight ≥ 2000 g. We found 81.4% neonates with severe respiratory failure responded to rescue HFOV treatment and significantly improved oxygenation was observed within 24–48 h after treatment, although the final mortality remained high. Besides, a simple and applicable prognostic scoring model with acceptable predictive power and accuracy was developed based on six independent risk factors. In addition to the initial severity of respiratory failure and response to HFOV, we found occurrence of sepsis and numbers of organ dysfunctions independently associated with final in-hospital mortality. Furthermore, none of the specific pulmonary diseases was independently associated with the outcomes.

Limited data about rescue use of HFOV in premature infants with severe respiratory failure can be found in the literature because it is difficult to define the “rescue” use of HFOV and conduct a randomized control trial^9,20,23,24^. Therefore, most studies have focused on comparisons between elective HFOV versus conventional ventilation^14–16,18^. We aimed to investigate the impacts of HFOV as the rescue use for critically ill preterm infants, so only patients poorly responded to conventional ventilation and those who met refractory respiratory failure were enrolled. We defined an initial OI ≥ 20 at initiation of HFOV as the rescue use because these patients were rarely successfully treated by conventional ventilation alone at the maximal ventilatory settings. Besides, the OI has a strong predictive power to reliably assess the severity of respiratory failure^30,31^. Patients with an initial OI < 20 on HFOV who experienced a rapid improvement or smooth treatment course after HFOV treatment were not enrolled since conventional ventilation can potentially be the alternative choice for these patients.

We found initial severity of respiratory and initial response to HFOV did contribute significantly to final mortality. Of note, the highest level of OI was not independently associated final mortality. It is also reasonable that hypoxia associated organ dysfunctions is independently associated with final mortality^32,33^. Therefore, the initial “golden period” of the most critically illness can have a significant impact on outcomes, which highlight the importance of initial therapeutic strategy to avoid organ dysfunctions. Although the AUC, sensitivity and specificity of this nomogram model are all less than 0.8, we still considered it satisfying because most neonatal severity scores cannot more accurately predict the final mortality^34,35^. Besides, some of these patients have prolonged HFOV use and hospitalization for more than 3 to 4 months, the chronic comorbidities and subsequent nosocomial infections after successful weaning from rescue HFOV may contribute to final outcomes.

A proportion of patients who received HFOV as early elective use experienced clinical deterioration 2 or 3 days later. The most important cause of clinical deterioration was sepsis, which contributed significantly to final in-hospital mortality in neonates with respiratory failure^36,37^. These patients were enrolled for analysis because they may eventually need HFOV support even though conventional ventilation was used at the maximal settings. Although these patients with clinical deterioration had a significantly higher mortality rate than those who well responded to HFOV at the first 24–48 h, they were selected by confounding factors of sepsis or other ventilation-associated events. Actually the mortality rates were comparable between premature infants who initially received elective use of HFOV and those who received rescue use of HFOV (27.0% versus 28.8%).

In this study, we analyzed a wide variety of patients (i.e., neonates with RDS, severe BPD, and secondary pulmonary hypertension) together. Similar to previous studies, we found none of specific pulmonary diseases was independently associated with the final outcome^38–40^. However, previous studies found mortality was not correlated with lower birth body weight, which may be due to different study population, different therapeutic interventions and various definition of respiratory failure^38–40^. Besides, our data also highlights the important role of initial-HFOV patient characteristics and aggressive treatment of sepsis to prompt neonates toward optimized outcomes.

Little data exist to guide early adjuvant therapy use in neonates with severe respiratory failure who receive HFOV. In out institute, iNO has been used as the off-label rescue regimen for preterm neonates with refractory respiratory failure with/without documented PPHN^24^. Because a proportion of preterm neonates presenting with severe hypoxemia during the immediate postnatal period is associated with acute pulmonary hypertension^38^, iNO has been proven effective as rescue therapy for these patients^24,38,41,42^. However, some patients received iNO due to BPD with secondary pulmonary hypertension and iNO was not effective in this subjects, which was compatible with previous studies that found later use of iNO is only effective to prevent BPD^43,44^. Recent systemic reviews have concluded that early routine use of iNO in preterm infants does not improve survival in preterm infants^43,44^. A randomized controlled trial would be needed to investigate the indication of initiating iNO in preterm infants on HFOV therapy.

There are several limitations in this study. First, the initiation of HFOV was based on the attending physician’s decision, and some patients with an initial OI between 15 and 20 were not enrolled because it was difficult to define whether their use of HFOV as rescue or elective. Second, this is a two-centers retrospective study. Some of the parameters were affected after partial treatment, and it is impossible to have a control group that used conventional ventilation at the most critical moment. Therefore, we cannot conclude the advantages of HFOV over conventional ventilation. Lastly, although we documented the independent risk factors for final mortality, the variables of some neonates who had a prolonged hospitalization were not collected and analyzed, which accounted for the less convincing predictive power of the nomogram model to predict final in-hospital mortality.

## Conclusion

HFOV is the most common and widely applicable therapeutic intervention for neonates with respiratory failure, especially in institutes without ECMO capability. In preterm neonates with severe refractory respiratory failure, the mortality rate remains high. We found the severity of respiratory failure, judged by initial OI, severity of hypotension and initial response to HFOV are significantly associated with final in-hospital mortality. Further efforts to avoid end-organ dysfunctions and aggressive treatment for sepsis are potentially feasible targets in order to optimize the outcomes.

## Supplementary Information

Supplementary Information.

## Data Availability

The datasets used/or analyzed during the current study available from the corresponding author on reasonable request.

## Abbreviations

HFOV: High-frequency oscillatory ventilation
IQR: Interquartile range
95% CI: 95% confidence interval
CGMH: Chang Gung Memorial Hospital
iNO: Inhaled nitric oxide
RDS: Respiratory distress syndrome
PPHN: Persistent pulmonary hypertension of newborn
BPD: Bronchopulmonary dysplasia
OI: Oxygenation index
OR: Odds ratio
CDH: Congenital diaphragmatic hernia
MAP: Mean airway pressure
