# Supplementary material for: Therapeutic effects and outcomes of rescue high-frequency oscillatory ventilation for premature infants with severe refractory respiratory failure
Source: Sci Rep. 2021 Apr 19;11:8471. doi: 10.1038/s41598-021-88231-6 (PMC8055989; doi:10.1038/s41598-021-88231-6)
Supplement: Supplementary file 1 — Supplementary Information. [file 41598_2021_88231_MOESM1_ESM.pdf]

The supplementary file

Title:

Therapeutic effects and outcomes of rescue high-frequency oscillatory ventilation for premature infants with severe refractory respiratory failure

Jen-Fu Hsu, MD[1,5]; Mei-Chin Yang, RT[2,3]; Shih-Ming Chu, MD[1,5]; Lan-Yan Yang, PhD[6]; Ming-Chou Chiang, MD[1,5]; Mei-Yin Lai, MD[1,5]; Hsuan-Rong Huang, MD[1,5]; Yu-Bin Pan, MS[6]; Ren-Huei Fu, MD PhD[1,5]; and Ming-Horng Tsai, MD PhD[4,5*]

Affiliations: [1]Division of Neonatology, Department of Pediatrics, Chang Gung Memorial Hospital, Taoyuan, Taiwan; [2]Department of respiratory therapy, Chang Gung Memorial Hospital, Taipei, Taiwan; [3]School of Business, Executive MBA program in Health Care Management, Chang Gung University, Taoyuan, Taiwan; [4]Division of Neonatology and Pediatric Hematology/Oncology, Department of Pediatrics, Chang Gung Memorial Hospital, Yunlin, Taiwan; [5]College of Medicine, Chang Gung University, Taoyuan, Taiwan; [6]Biostatistics Unit of Clinical Trial Center, Chang Gung Memorial Hospital, Linkou, Taiwan

***Address correspondence to:** Dr. Ming-Horng Tsai, MD, PhD.

Division of Neonatology and Pediatric Hematology/Oncology, Department of Pediatrics, Chang Gung Memorial Hospital, Yunlin, Taiwan;

No.707, Gongye Rd., Sansheng, Mailiao Township, Yunlin, Taiwan, R.O.C.

Phone: 886-5-6915151 ext. 2893; Fax: 886-5-691-3222

E-mail: mingmin.tw@yahoo.com.tw

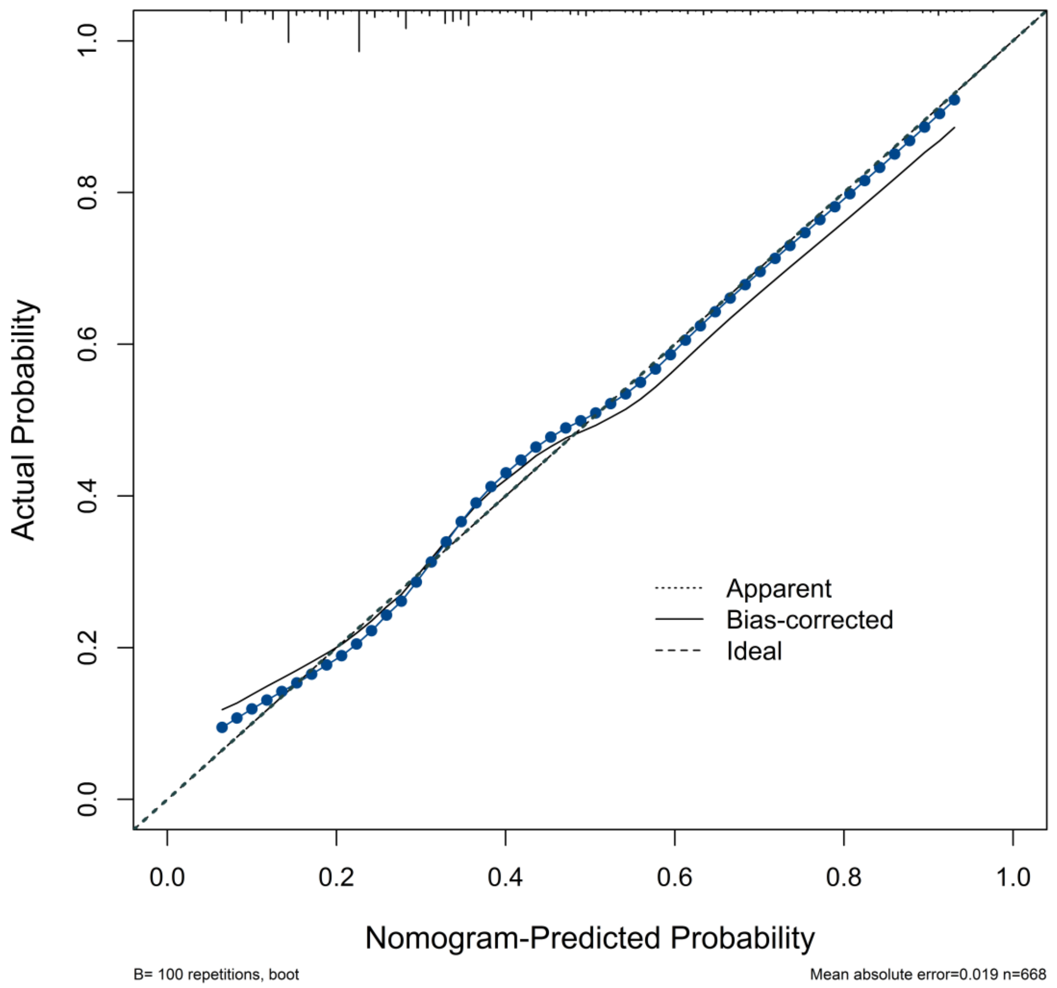

Supplementary Figure 1.

Receive operating characteristic (ROC) plots. The area under the ROC curve (AUC) value was 0.768 for the formulated nomogram for the prediction of in-hospital mortality

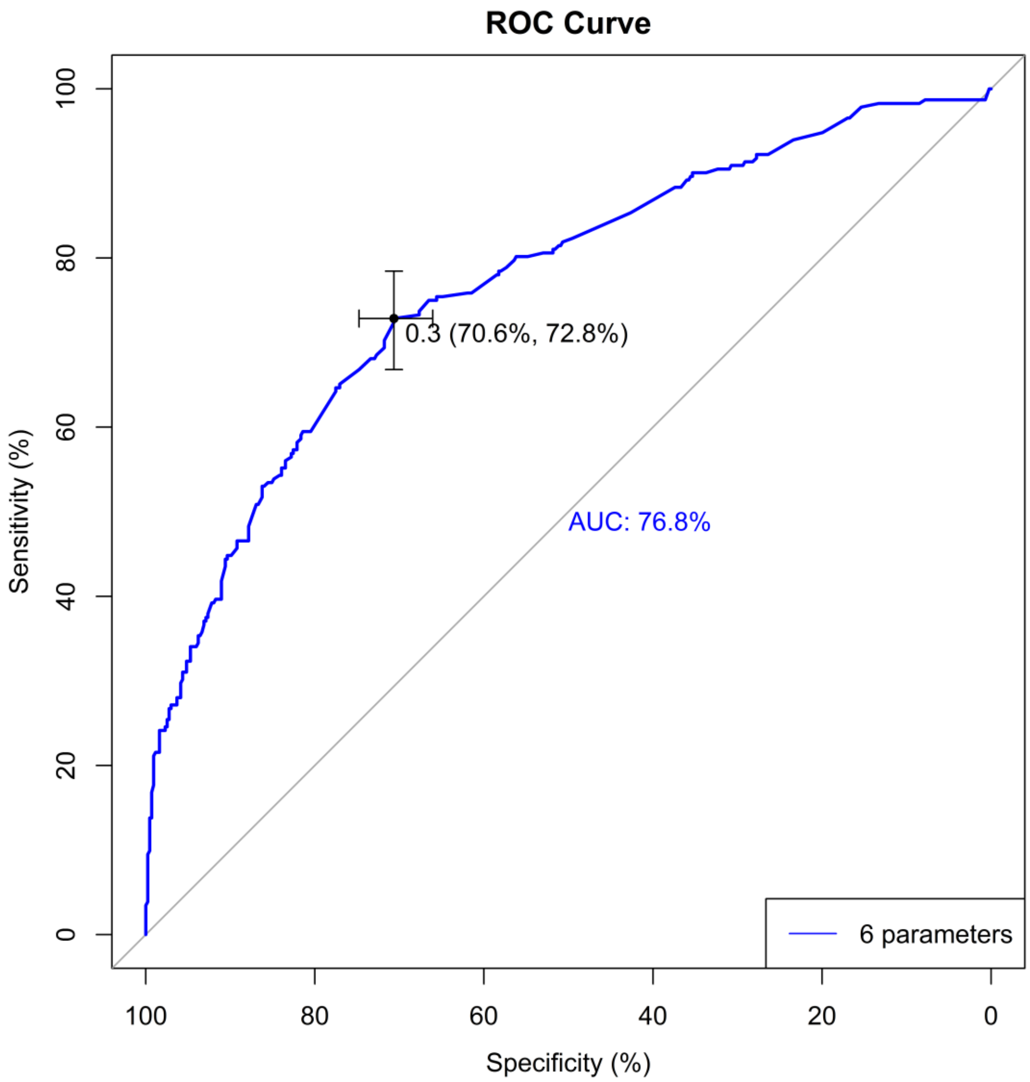

Supplementary Figure 2.

The calibration curve for the prediction model. The nomogram-predicted probabilities of in-hospital mortality were similar to the actual probabilities of in-hospital mortality

Supplementary Table 1. Criteria to define severity of hypotension and four major organ dysfunction syndrome criteria

Severity of hypotension	Major Organ Dysfunction
*Mild hypotension: patients who required only one cardioinotropic agents (usually dopamine < 10ug/kg/min)	*Neurological dysfunction: altered level of central nervous system or seizure
*Moderate hypotension: patients who required both dopamine and dobutamine, with only one of them ≥ 10ug/kg/min to maintain adequate blood pressure	*Renal dysfunction: BUN/C4 creatinine level > 30<br>*Hepatic: bilirubin > 3.5 mg/dL in the first 72 hours<br>And: ALT > 72 "or" maximum value
*Severe hypotension: patients who required epinephrine and/or more than two cardioinotropic agents (usually dopamine and dobutamine, both ≥ 10ug/kg/min) to maintain adequate blood pressure	And: AST > 92 "or" maximum value in the first 72 hour<br>*Hematologic: platelet count < 50,000/μl<br>Or: prolonged PT and aPTT

Supplementary Table 2. Risk factors for overall mortality by univariate and multivariate analysis

Risk factor	Univariate analysis			Multivariate analysis	
	Survived, N=436, n (%)	Died, N=232, n (%)	*P* value	Adjusted OR (95% CI)	*P* value
Gestational age (weeks)			0.052		
< 28 weeks	223 (51.1)	141 (60.8)		2.16 (1.24-3.75)	0.007
28-31 weeks	111 (25.5)	50 (21.6)		1.36 (0.77-2.41)	0.450
≥ 32 weeks	102 (23.4)	41 (17.7)		1 (reference)	
Low apgar score at 5 minutes (≤ 7)	226 (51.8)	158 (68.1)	0.015	1.22 (0.68-2.19)	0.512
Perinatal asphyxia	35 (8.0)	33 (14.2)	0.001	1.04 (0.48-2.24)	0.919
Occurrence of sepsis	67 (15.4)	68 (29.3)	< 0.001	1.82 (1.17-2.85)	0.008
Highest OI during HFOV treatment			< 0.001		
≤ 30	298 (68.3)	105 (45.3)		1 (reference)	
31-40	57 (13.1)	27 (11.6)		0.97 (0.52-1.64)	0.746
41-50	28 (6.4)	23 (9.9)		1.25 (0.62-2.55)	0.533
> 50	53 (12.2)	77 (33.2)		1.44 (0.83-2.49)	0.190
Initial OI at HFOV treatment※			< 0.001		
≤ 20	364 (83.5)	146 (62.9)		1 (reference)	
21-30	52 (11.9)	27 (11.6)		0.73 (0.38-1.39)	0.336
31-40	11 (2.5)	15 (6.5)		0.93 (0.37-2.55)	0.880
> 40	9 (2.1)	44 (19.0)		3.31 (1.27-8.66)	0.015
Response to HFOV within the first 3 days			< 0.001		
Good response	315 (72.2)	110 (47.4)		1 (reference)	

Non-significant improvement	82 (18.8)	36 (15.5)		1.29 (0.77-2.17)	0.334
Failure	39 (8.9)	86 (37.1)		4.05 (2.08-7.90)	< 0.001
Number of organ dysfunction*			< 0.001		
no	116 (26.6)	32 (13.8)		1 (reference)	
1	188 (43.1)	90 (38.8)		1.77 (1.01-3.08)	0.045
2	49 (11.2)	37 (15.9)		2.45 (1.34-4.47)	0.004
≥ 3	83 (19.0)	73 (31.5)		3.05 (1.55-5.98)	0.001
Severity of hypotension**			< 0.001		
No	146 (33.5)	51 (22.0)		1 (reference)	
Mild hypotension	154 (35.3)	55 (23.7)		1.01 (0.62-1.63)	0.992
Moderate hypotension	116 (26.6)	83 (35.8)		1.91 (1.17-3.09)	0.009
Severe hypotension	20 (4.6)	43 (18.5)		4.37 (2.17-8.80)	< 0.001
Primary pulmonary diseases					
Severe RDS[#]	156 (35.8)	82 (35.3)	0.506		
PPHN	87 (20.0)	62 (26.9)	0.051	1.44 (0.88-2.37)	0.151
Meconium aspiration syndrome	12 (2.8)	3 (5.8)	0.226		
Ventilator-associated pneumonia	10 (2.3)	7 (53.8)	0.610		
Pulmonary hemorrhage	33 (7.6)	32 (23.1)	0.013	1.58 (0.88-2.76)	0.129
Pneumothorax	37 (8.5)	24 (5.8)	0.481		
Secondary pulmonary hypertension	2 (0.5)	11 (11.5)	< 0.001	3.75 (0.73-19.32)	0.115
Congenital diaphramatic hernia	6 (1.4)	5 (3.8)	0.527		
Presences of comorbidities					

Intraventricular hemorrhage[&]	37 (8.5)	26 (11.2)	0.268		
Patent ductus arteriosus	206 (47.2)	83 (35.8)	0.005	0.83 (0.56-1.22)	0.344

OI: oxygenation index; RDS: respiratory distress syndrome; PPHN: persistent pulmonary hypertension of newborn; OR: odds ratio, 95% CI: 95% confidence interval

※The average oxygenation index at 2 and 6 hours after initiation of HFOV was used

*Number of organ dysfunction: including neurological, renal, hematological, and hepatic dysfunction

**Mild hypotension was defined as patients who required only one cardioinotropic agents (usually dopamine < 10ug/kg/min)

**Moderate hypotension was defined as patients who required both dopamine and dobutamine, with only one of them ≥ 10ug/kg/min to maintain adequate blood pressure

**Severe hypotension was defined as patients who required epinephrine and/or more than two cardioinotropic agents (usually dopamine and dobutamine, both ≥ 10ug/kg/min) to maintain adequate blood pressure

#Severe RDS was defined as patients who required ≥ 2 doses of surfactant

[&]Intraventricular hemorrhage > = grade III
